# Identification of the causal relationship between sleep quality, insomnia, and oral ulcers

**DOI:** 10.1186/s12903-023-03417-w

**Published:** 2023-10-13

**Authors:** Qianxi Liu, Jiongke Wang, Tiannan Liu, Xin Zeng, Xuefeng Zhang

**Affiliations:** 1 State Key Laboratory of Oral Diseases, Research Unit of Oral Carcinogenesis and Management, West China Hospital of Stomatology, National Clinical Research Center for Oral Diseases, Chinese Academy of Medical Sciences, Sichuan University, 3rd Section of Ren Min Nan Rd, Chengdu, 610041 Sichuan P. R. China; 2 Emergency Department, State Key Laboratory of Oral Diseases, National Clinical Research Center for Oral Diseases, Research Unit of Oral Carcinogenesis and Management, West China Hospital of Stomatology, Chinese Academy of Medical Sciences, Sichuan University, Chengdu, 610041 Sichuan P. R. China

**Keywords:** Sleep duration, Insomnia, Mouth ulcer, Mendelian randomization, Genetic correlation

## Abstract

**Background:**

Multiple epidemiological studies have posited a potential association between sleep quality and the risk of oral diseases, yet the resulting conclusions have remained contentious, and the presence of a causal link remains equivocal. In this study, we aimed to investigate the causal relationship between sleep duration, insomnia, and common oral diseases.

**Methods:**

We utilized genetic correlation and two-sample Mendelian randomization analyses based on summary statistics from genome-wide association studies of sleep duration (N = 460,099), insomnia (N = 462,341), mouth ulcer (N = 385,026), oral cavity cancer (N = 4,151), and periodontal disease (N = 527,652).

**Results:**

Our results revealed a negative genetic correlation between sleep duration and mouth ulcer (genetic correlation: -0.09, P = 0.007), while a positive genetic correlation between insomnia and mouth ulcer was observed (genetic correlation: 0.18, P = 2.51E-06). Furthermore, we demonstrated that longer sleep duration is significantly associated with a reduced risk of mouth ulcers (OR: 0.67, 95% CI: 0.54–0.83, P = 2.84E-04), whereas insomnia is nominally associated with an increased risk of mouth ulcers (OR: 1.40, 95% CI: 1.01–1.95, P = 0.044). In contrast, no significant association was detected between sleep quality and periodontal disease or oral cavity cancer.

**Conclusions:**

This work provides robust evidence to support the notion that enhanced sleep quality may confer a decreased risk of oral ulcers, thereby bearing considerable clinical relevance.

**Supplementary Information:**

The online version contains supplementary material available at 10.1186/s12903-023-03417-w.

## Introduction

Mouth ulcers are common oral disease, affecting around 20% of the population [1]. Though mostly mouth ulcers are harmless and clear up on their own, they impaired the oral quality of life and vitality. In addition to some cases that can be attributed to local stimulus like mechanical or physical factors, most oral ulcers occur due to the combination of both local and systemic causes [2]. From the etiology perspective, causes of oral ulcers are related to traumatic, infectious, allergic factors, and may be associated with skin disease, autoimmune disease, tumor, and inflammatory bowel disease [3]. Though the exact pathogenesis is largely unknown, multiple factors could contribute to the risk of mouth ulcers, such as family history, less physical exercise, and inadequate brushing time [1]. Identification of risk factors for mouth ulcers can help defining effective strategies to monitor, prevent and control the disease.

Sleep is a fundamental process which regulates core biological functions of the body, and was shown to play a key role in oral health as well [4]. For example, previous epidemiological study among 62,276 adolescents found that low quality of sleep was associated with a higher risk of oral symptoms like toothache [5]. Another cross-sectional study within 29,870 individuals from the French population demonstrated that individuals with sleep disorders were at higher risk of gingival inflammation (OR: 1.22, 95% CI: 1.13–1.32) [6]. Pathologically, disrupted sleep has been linked to elevated levels of stress, depression, anxiety, and fatigue [7], all of which may increase the susceptibility to oral diseases [8, 9]. Meanwhile, sleep deprivation is known to induce oxidative stress, a key contributor to the development of oral diseases [10]. Additionally, sleep deprivation was found to be associated with temporomandibular joint disorder (TMJ) and bruxism [11], while these two disorders were risk factors for oral ulcers as well. The lack of sleep may increase the body’s risk of inflammation, and cause fatigue and stress, thus causing TMJ and bruxism. Meanwhile, TMJ and bruxism were also shown to have a negative impact on children’s sleeping habits and characteristics [11], though inconsistent results have also been reported [12]. All these observations strongly support the notion that sleep quality is closely associated with oral health. However, whether sleep quality causally influences the development of mouth ulcers remains elusive. Moreover, observational studies face the challenge of addressing confounding biases without randomization, making it difficult to establish causation.

Mendelian randomization (MR) is a useful causal inference method which could avoid common pitfalls in observational studies. The MR method uses genetic variation to infer causal association between modifiable exposures and health, developmental or social outcomes [13]. By utilizing genetic variants as instrumental variables, the MR method provides a means to mitigate the impact of unobserved confounding factors on the relationship between exposure and outcome.

Here, the aim of our study was to examine the causal relationship between sleep duration, insomnia, and the incidence of common oral diseases, specifically mouth ulcers, periodontal disease, and oral cavity cancer, using the two-sample MR method. Our findings demonstrated a significant association between longer sleep duration and a lower risk of mouth ulcers, whereas a nominal association was identified between insomnia and increased risk of mouth ulcers.

## Methods

### Data source

The GWAS summary datasets used in this study were listed in **Supplementary Table **1. Summary statistics of sleep duration were from a genome-wide association study (GWAS) based on the UK Biobank data (N = 460,099). Sleep duration was measured by “About how many hours sleep do you get in every 24 hours” in the UK Biobank (data field: 1160). Summary statistics of insomnia were from GWAS based on the UK Biobank data (N = 462,341). Insomnia was measured by “Do you have trouble falling asleep at night or do you wake up in the middle of the night?” in the UK Biobank (data field: 1200). A detailed description of the study design, including quality control procedures and statistical analyses, is available at http://www.nealelab.is/uk-biobank/. We analyzed three oral diseases as outcomes, including mouth ulcer (N = 385,026) [14], periodontal disease (N = 527,652) [15], and oral cavity cancer (N = 4,151) [16], based on available summary statistics from previous GWAS. Single nucleotide polymorphisms (SNP) with genome-wide significance threshold (P < 5E-08) and in low linkage-disequilibrium with other SNPs (r^2^ < 0.001) within a clumping distance of 10,000 kb in each trait as exposure were selected as instrumental variables. Effects are harmonized to ensure that the effect estimates in both the exposure and outcome GWAS correspond to the same allele for each SNP.

### Genetic correlation

Genetic correlation is a measure of association between the genetic determinants of two phenotypes, as well as an informative metric to quantify the overall genetic similarity between complex traits, which provides insights into their polygenic genetic architecture. Identifying genetic correlation between complex traits can provide useful etiological insights and help prioritize likely causal relationships. In the current study, we applied the LD-score regression method (LDSC) to estimate the genetic correlation between sleep duration, insomnia, and oral diseases [17]. Briefly, this method generates a score reflecting whether the GWAS test statistic of a biologically relevant variant correlates with nearby variants in high linkage disequilibrium. The z statistic for the genetic association of each variant with the first trait are multiplied with the z statistic for the genetic association with the second trait, followed by regression of this product of statistics against the LD scores. The slope (coefficient) represents genetic correlation. The reference data was derived from the 1000 Genomes Project European population. A P value below 8.33E-03 (0.05/6) was considered statistically significant after the Bonferroni correction.

### Two-sample mendelian randomization analysis

Three essential assumptions are prerequisites for conducting MR: (A) the relevance assumption, the instrumental variables are associated with the exposure; (B) the independence assumption, the instrumental variables are independent of any confounding factors of the exposure-outcome association; and (C) the exclusion-restriction assumption, the instrumental variables are conditionally independent of the outcome given the exposure and the confounding factors.

To evaluate the causative effect of sleep duration and insomnia on the risk of oral diseases, we performed a two-sample MR analysis using the random effects inverse variance weighted method. Bonferroni-corrected thresholds (0.05/6 = 8.33E-03) were adopted to account for multiple testing. We further verified the significant results using weighted median and weighted mode methods. To assess whether the MR assumptions were violated in the analysis, we conducted a number of sensitivity analyses. We calculated the F-statistic of each SNP, which could reflect the exact strength of the effect of SNPs on the exposure traits. SNPs with F statistic below 10 were considered as weak instruments and were thus removed. Multiple SNPs selected as instrumental variables are inevitably subjected to the pleiotropy issue. We utilized MR-PRESSO to test for horizontal pleiotropic outliers, and removed the outliers to reduce the effect of horizontal pleiotropy. Cochran’s Q statistic, which is derived from the IVW estimate, should follow a χ2 distribution with degrees of freedom equal to the number of SNPs minus 1. We performed Cochran’s Q test to check heterogeneity in the MR estimates. We further applied MR-Egger regression, a weighted linear regression of the SNP-outcome effects on the SNP-exposure effects allowing for the intercept to be estimated. The intercept provides a measure of average pleiotropic bias. The statistical power was calculated at http://cnsgenomics.com/shiny/mRnd/. The R package TwoSampleMR 0.5.6 was used for the statistical analyses.

## Results

We first estimated the genetic correlation between sleep duration, insomnia, and oral diseases. We detected a significant and negative genetic correlation between sleep duration and mouth ulcers (genetic correlation: -0.09, SE = 0.03, P = 0.007). Meanwhile, a significant and positive genetic correlation was identified between insomnia and mouth ulcers (genetic correlation: 0.18, SE = 0.04, P = 2.51E-06) (**Supplementary Fig. **1).

**Fig. 1:**
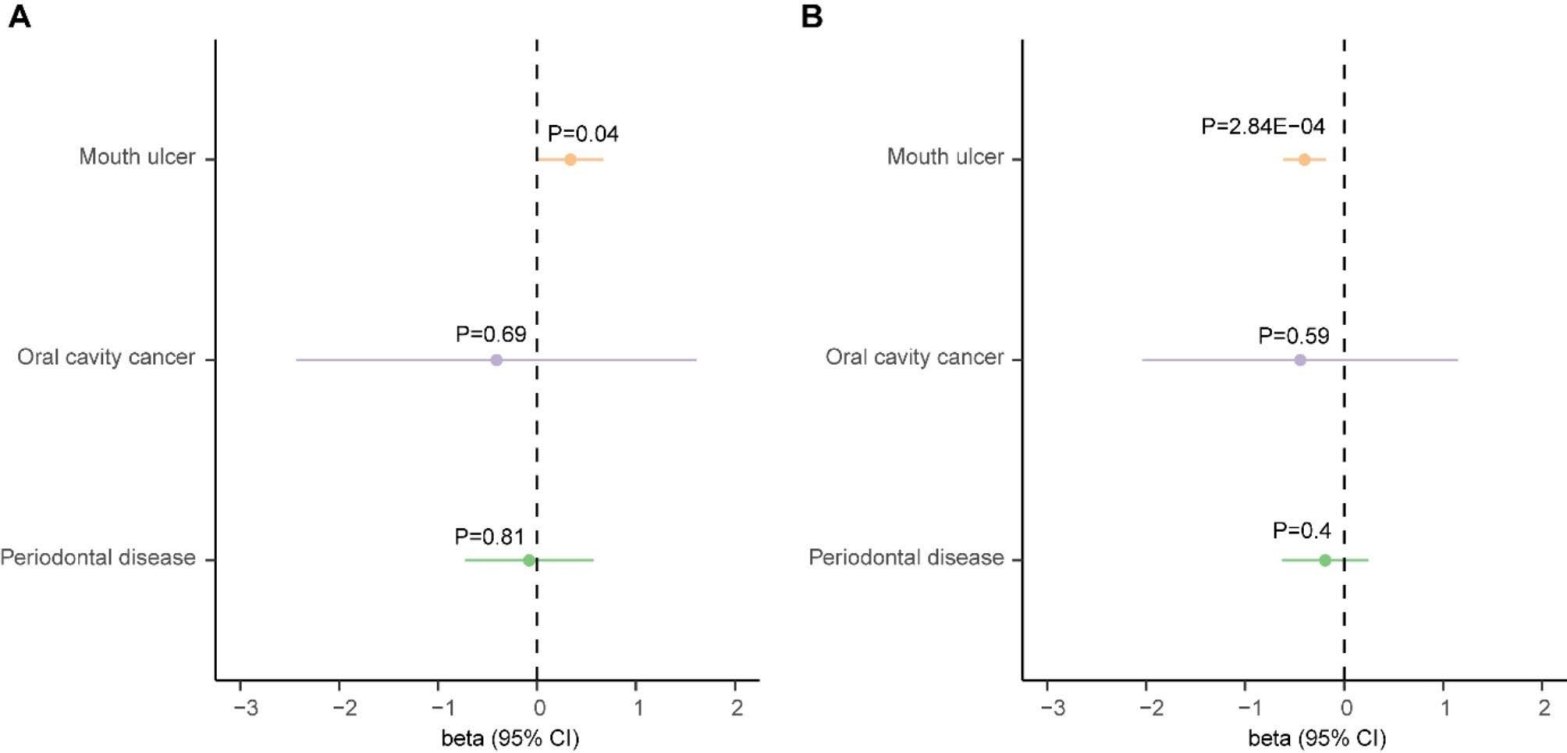
Forest plot showing results from the Mendelian randomization analysis Results from the Mendelian randomization (MR) analysis to evaluate causal role of **(A)** sleep duration, and **(B)** insomnia on oral manifestations using the inverse variance weighted method. Estimates are per 1 standard deviation (SD) increase in the trait

**Fig. 2:**
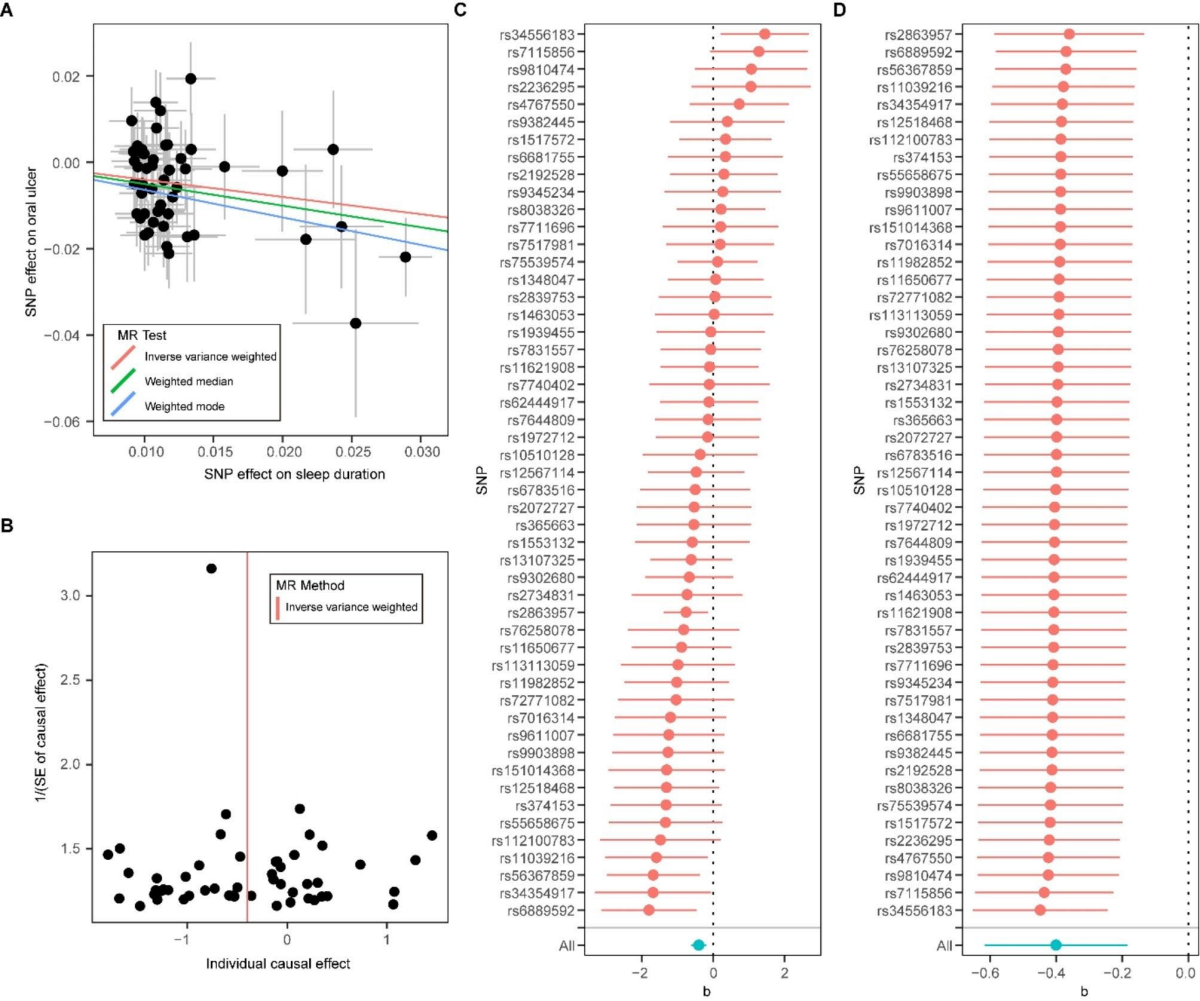
Mendelian randomization analysis results for sleep duration on risk of mouth ulcer **(A)** Scatter plot of genetic associations with sleep duration (horizontal lines) against genetic associations with mouth ulcer (vertical lines). Error bars for genetic associations are 95% confidence intervals. The slopes of each line in the scatter plot represent the causal association for each method. **(B)** Funnel plot of single-SNP effect estimates and corresponding inverse standard errors. **(C)** Forest plot of the association of individual SNPs with sleep duration and mouth ulcer, together with pooled estimates. **(D)** Forest plot of the results of the leave-one-out sensitivity analysis, where each SNP in the instrument was iteratively removed from the instrumental variables

We then analyzed the role of sleep duration and insomnia in the risk of three common oral diseases via the two-sample MR approach. Results showed that one standard deviation increase in genetically determined sleep duration was associated with a reduced risk of mouth ulcers (OR:0.67, 95% CI:0.54–0.83, P = 2.84E-04) (Fig. 1). Such association was further verified by the weighted median (OR: 0.61, 95% CI: 0.44–0.83, P = 1.60E-03) and weighted mode (OR: 0.53, 95% CI: 0.30–0.95, P = 0.037) methods. The funnel plot showed a visually apparent symmetry, which excluded the possible influence of directional pleiotropy on our estimates (Fig. 2). Consistent with this finding, insomnia was nominally associated with an increased risk of mouth ulcers (OR:1.40, 95% CI:1.01–1.95, P = 0.044) (Fig. 1). However, the weighted median and weighted mode methods did not detect association. In contrast, we did not identify association between sleep duration, insomnia and periodontal disease, or oral cavity cancer (**Supplementary Figs. **2–6).

Furthermore, we performed a number of sensitivity analyses to validate the causal association between sleep duration and the risk of oral diseases. No heterogeneity of effects between the instrumental variables was detected by the Cochran’s Q test (**Supplementary Table **2). The F statistics of all the instrument variables were above 10 (ranging from 29 to 224), suggesting the selected instrumental variables were strong enough. The MR-Egger regression analysis gave no significant evidence of horizontal pleiotropy, as the intercept was not significantly deviated from zero (**Supplementary Table **2). Meanwhile, no potential instrumental outlier was detected by the MR-PRESSO analysis (**Supplementary Table **2).

## Discussion

A number of risk factors have been identified to contribute to the development of oral manifestations. For example, one previous study among 156 adult individuals observed a significantly lower resting flow rate of saliva and significantly higher prevalence of xerostomia in patients with type 1 diabetics compared to healthy subjects [18]. SAPHO syndrome, a rare disease affecting the joints, bones and skin, might present some orofacial manifestations as well, mainly mandibular osteomyelitis [19]. In addition, oral manifestations were also frequently reported in infectious diseases such as monkeypox and HIV [20, 21]. Meanwhile, several studies in the literature have reported that patients infected with COVID-19 or following vaccine administration showed different manifestations of orofacial signs and symptoms such as ulceration and periodontal disease [22, 23], though inconsistent results have been reported [24]. These multiple risk factors from different aspects suggested the complex etiology of the oral diseases, and also posted an urgent need to explore unestablished risk factors to better understand the pathogenesis of the diseases. The potential association between sleep duration and oral diseases has been suggested in previous clinical and epidemiological studies, but the results have been inconsistent. Moreover, adjusting for confounding factors is challenging in observational studies, which could introduce bias. In this study, we investigated the causal role of sleep duration and insomnia in the risk of three oral diseases (mouth ulcer, periodontal disease, and oral cavity cancer) using the MR approach. Our results showed that higher sleep duration was significantly associated with a reduced risk of mouth ulcers, whereas insomnia was nominally associated with an increased risk of mouth ulcers. These findings provide a better understanding of the role of sleep in oral diseases and offer lifestyle recommendations to individuals prone to developing mouth ulcers.

A correlation between good sleep and improved oral health, such as a decreased risk of mouth ulcers, has been well-documented. An earlier experimental study has demonstrated that sleep deprivation resulted in exacerbation of oral ulcers and a delay in their healing process in a rat model [1]. One possible reason is that sleep duration could affect mouth ulcers by modulating immunologic response and inflammatory mediators [25]. It was suggested that sleep deprivation increased serum TNF-α, IL-1β, IL-6, IL-8, and MCP-1 levels [10], while excessive production of TNF-α, IL-1β, and IL-6 were associated with an increased risk of recurrent aphthous stomatitis [26]. Additionally, sleep deprivation induces oxidative stress in the body, which plays an important role in the oral mucosal disease pathogenesis [27]. Therefore, inflammation and oxidative stress might be involved in the pathogenesis of how short sleep duration increased the risk of mouth ulcers. Notably, it was also reported that both short and long sleep durations were significantly associated with the development of poor oral health status [28, 29]. Thus, subsequent investigations of sleep duration and mouth ulcers may benefit from examining the impact of both insufficient and excessive sleep levels.

Previous studies also demonstrated association between sleep duration and periodontal disease, though the results were still conflicting [30]. Meanwhile, excessively short or long sleep duration was also shown to be associated with risk of several cancers [31]. In this study, we did not establish a causal link between sleep duration and the risk of periodontal disease and oral cavity cancer. This could be attributed to the possibility that the effect of sleep on these diseases is comparatively modest, or the underlying mechanism is distinct from that of mouth ulcers. Nevertheless, the degree of variance accounted for by the instrumental variables of the exposures was relatively limited, thus constraining the statistical power to detect associations with moderate effects (**Supplementary Table **3). Future analyses based on summary statistics from GWAS with larger sample sizes are imperative to furnish a more precise and reliable estimation.

The current study analyzed the role of sleep quality in common oral diseases. The MR approach was less susceptible to confounding or reverse causation. The large sample size of the GWAS also increased the statistical power. While our study made efforts to mitigate confounding bias through various MR methods and extensive sensitivity analyses, it is important to acknowledge certain limitations. Firstly, despite our selection of genetic variants from large-scale studies, we cannot completely rule out the possibility of weak instrument bias, as is common in all MR analyses. Second, the results were obtained from Caucasian individuals and might not be generalized to other ethnic populations. Further replication based on cohorts of different ancestry was still warranted. Third, even though two-sample MR can be performed when the exposure of interest and the outcome are not simultaneously measured within one dataset, full datasets such as large-scale patient cohorts such as discovery and external validation sets are needed for a comprehensive understanding of causality, considering potential confounding factors. Fourth, the insomnia diagnosis and sleep time were collected based on the individuals’ subjective measurement, which might not be accurate and thus influence the results.

## Conclusion

In summary, our study revealed that prolonged sleep duration was linked with a decreased risk of mouth ulcers, whereas insomnia was associated with an increased risk of mouth ulcers. These findings have implications for informing therapeutic interventions and drug development in future clinical trials.

### Electronic supplementary material

Below is the link to the electronic supplementary material.

**Supplementary Figure 1**. Forest plot showing results from the genetic correlation analysis. **Supplementary Figure 2**. MR analysis results for sleep duration on risk of oral cavity cancer. **Supplementary Figure 3**. MR analysis results for sleep duration on risk of periodontal disease. **Supplementary Figure 4**. MR analysis results for insomnia on risk of mouth ulcer. **Supplementary Figure 5**. MR analysis results for insomnia on risk of oral cavity cancer. **Supplementary Figure 6**. MR analysis results for insomnia on risk of periodontal disease. **Supplementary Table 1**. Summary data from all GWAS used in current study. **Supplementary Table 2**. Heterogeneity and horizontal pleiotropy analyses between insomnia, sleep duration and oral manifestations. **Supplementary Table 3**. Effect sizes can be detected with the power of 0.8 given the sample size, proportion of cases and variance explained by the instrumental variables.

Supplementary Material 2

## Data Availability

Summary statistics of sleep duration and insomnia could be downloaded from ieu open gwas project (https://gwas.mrcieu.ac.uk/, ID: ukb-b-4424 and ukb-b-3957). Summary statistics of periodontal disease and oral cavity cancer could be found in GWAS Catalog (ID: GCST90018897 and GCST012237). Summary statistics of mouth ulcer could be found in GWAS Atlas (https://atlas.ctglab.nl/traitDB/3544, atlas ID: 3544).
