# Supplementary material for: Identification of the causal relationship between sleep quality, insomnia, and oral ulcers
Source: BMC Oral Health. 2023 Oct 13;23:754. doi: 10.1186/s12903-023-03417-w (PMC10571295; doi:10.1186/s12903-023-03417-w)
Supplement: Supplementary file 1 — Supplementary Figure 1. Forest plot showing results from the genetic correlation analysis. Supplementary Figure 2. MR analysis results for sleep duration on risk of oral cavity cancer. Supplementary Figure 3. MR analysis results for sleep duration on risk of periodontal disease. Supplementary Figure 4. MR analysis results for insomnia on risk of mouth ulcer. Supplementary Figure 5. MR analysis results for insomnia on risk of oral cavity cancer. Supplementary Figure 6. MR analysis results for insomnia on risk of periodontal disease. Supplementary Table 1. Summary data from all GWAS used in current study. Supplementary Table 2. Heterogeneity and horizontal pleiotropy analyses between insomnia, sleep duration and oral manifestations. Supplementary Table 3. Effect sizes can be detected with the power of 0.8 given the sample size, proportion of cases and variance explained by the instrumental variables. [file 12903_2023_3417_MOESM1_ESM.docx]

**Supplementary Materials of**

**Identification of the Causal Relationship between Sleep Quality, Insomnia, and Oral Ulcers**

**Authors:** Qianxi Liu^1^, Jiongke Wang^1^, Tiannan Liu^1^, Xin Zeng^1,*^, Xuefeng Zhang^2,*^

**Affiliation:**

^1^ State Key Laboratory of Oral Diseases, National Clinical Research Center for Oral Diseases, Research Unit of Oral Carcinogenesis and Management, Chinese Academy of Medical Sciences, West China Hospital of Stomatology, Sichuan University, Chengdu, Sichuan 610041, P. R. China.

^2^ Emergency Department, State Key Laboratory of Oral Diseases, National Clinical Research Center for Oral Diseases, Research Unit of Oral Carcinogenesis and Management, Chinese Academy of Medical Sciences, West China Hospital of Stomatology, Sichuan University, Chengdu, Sichuan 610041, P. R. China.

***Correspondance:** Xin Zeng (zengxin@scu.edu.cn) and Xuefeng Zhang (zhangxuefeng1990@scu.edu.cn), No. 14, 3rd Section of Ren Min Nan Rd., West China Hospital of Stomatology, Sichuan University, Chengdu, 610041, China.

**This supplementary file includes,**

Supplementary Figure 1-6

Supplementary Table 1-3

**Supplementary Figures**


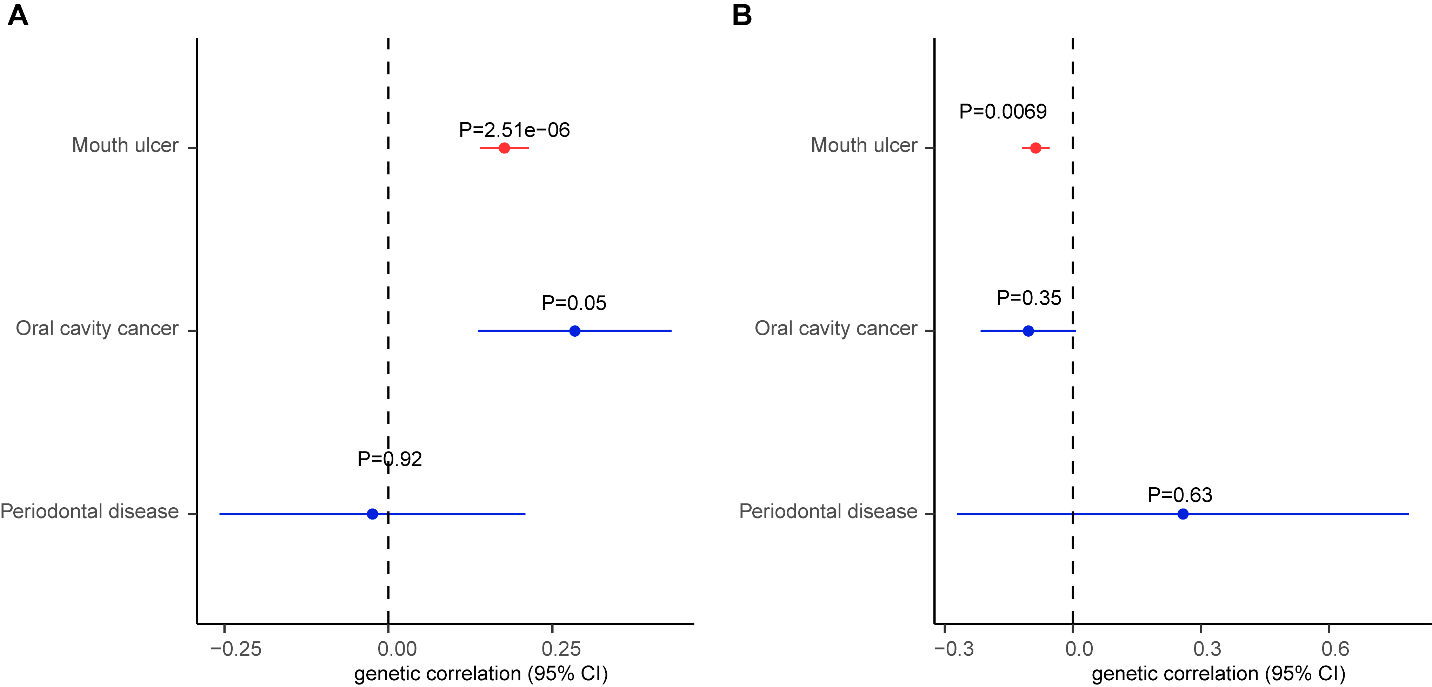
**Supplementary Figure 1. Forest plot showing results from the genetic correlation analysis.**

**(A)** Genetic correlation results between insomnia and oral manifestations.

**(B)** Genetic correlation results between sleep duration and oral manifestations. Error bars indicate 95% confidence intervals.


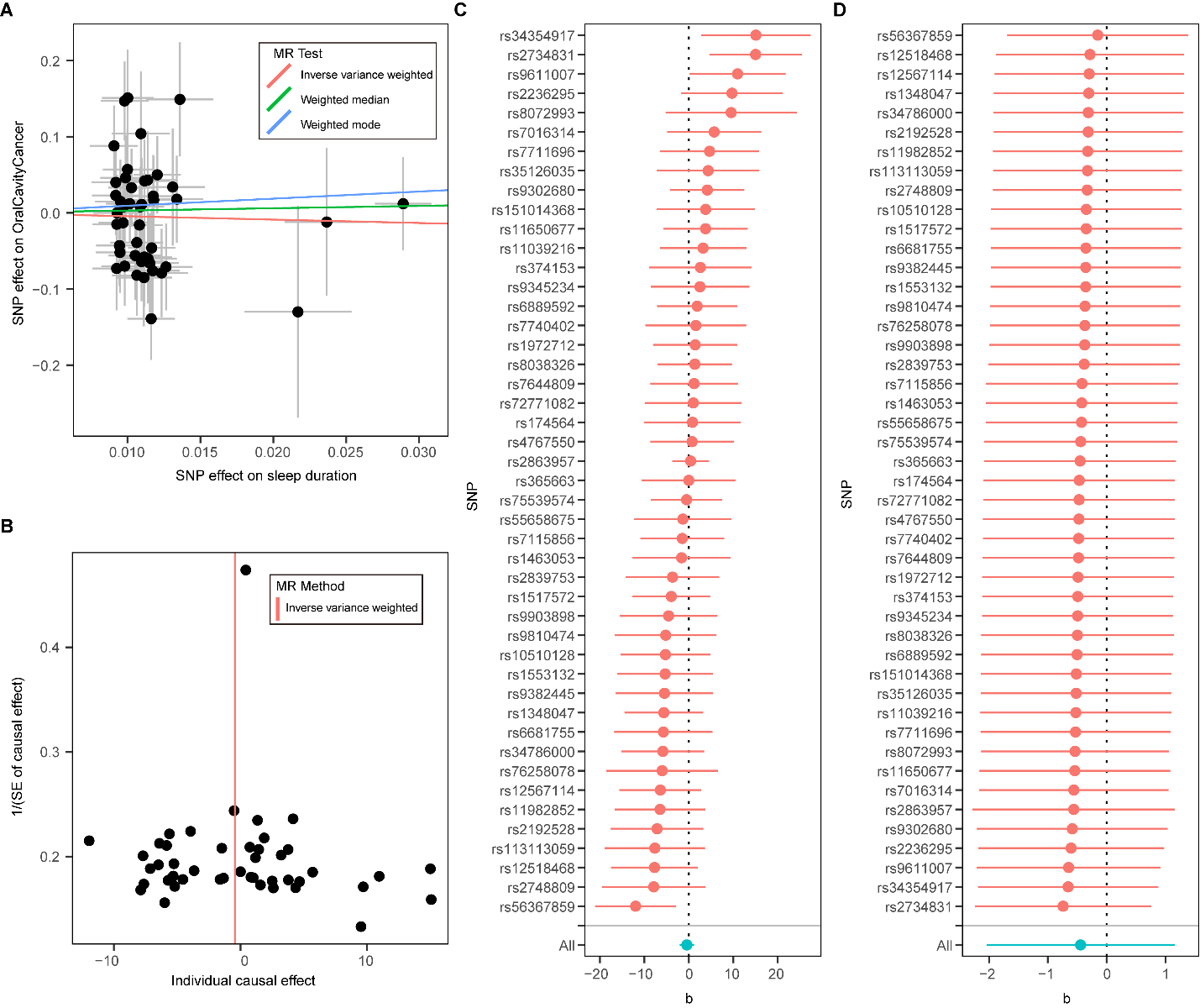
**Supplementary Figure 2. MR analysis results for sleep duration on risk of oral cavity cancer.**

**(A)** Scatter plot of genetic associations with sleep duration (horizontal lines) against genetic associations with oral cavity cancer (vertical lines). Error bars for genetic associations are 95% confidence intervals. The slopes of each line in the scatter plot represent the causal association for each method.

**(B)** Funnel plot of single-SNP effect estimates and corresponding inverse standard errors.

**(C)** Forest plot of the association of individual SNPs with sleep duration and oral cavity cancer, together with pooled estimates.

**(D)** Forest plot of the results of the leave-one-out sensitivity analysis, where each SNP in the instrument was iteratively removed from the instrumental variables.

**Supplementary Figure 3. MR analysis results for sleep duration on risk of** **periodontal disease.**


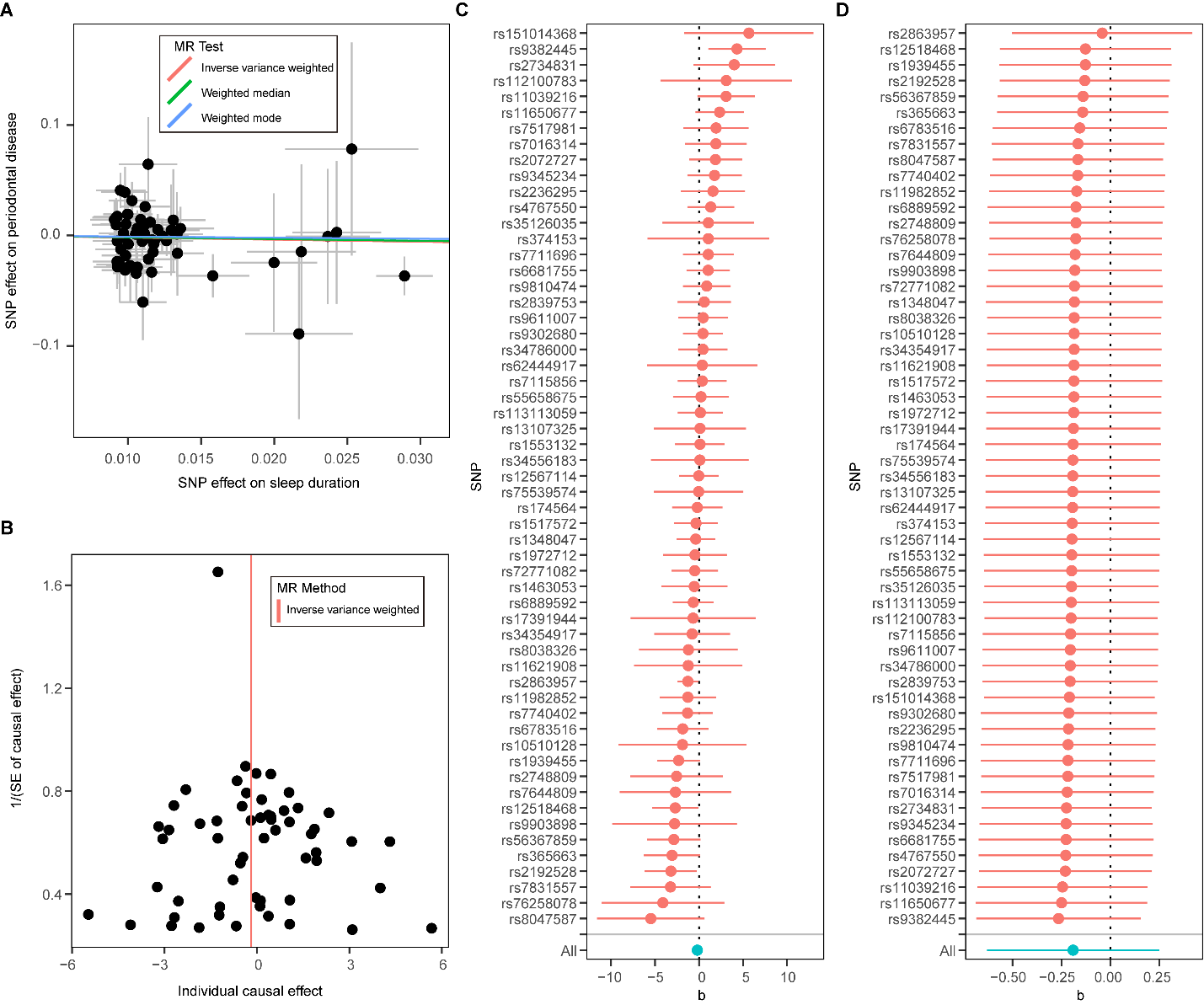


**(A)** Scatter plot of genetic associations with sleep duration (horizontal lines) against genetic associations with periodontal disease (vertical lines). Error bars for genetic associations are 95% confidence intervals. The slopes of each line in the scatter plot represent the causal association for each method.

**(B)** Funnel plot of single-SNP effect estimates and corresponding inverse standard errors.

**(C)** Forest plot of the association of individual SNPs with sleep duration and oral cavity cancer, together with pooled estimates.

**(D)** Forest plot of the results of the leave-one-out sensitivity analysis, where each SNP in the instrument was iteratively removed from the instrumental variables.


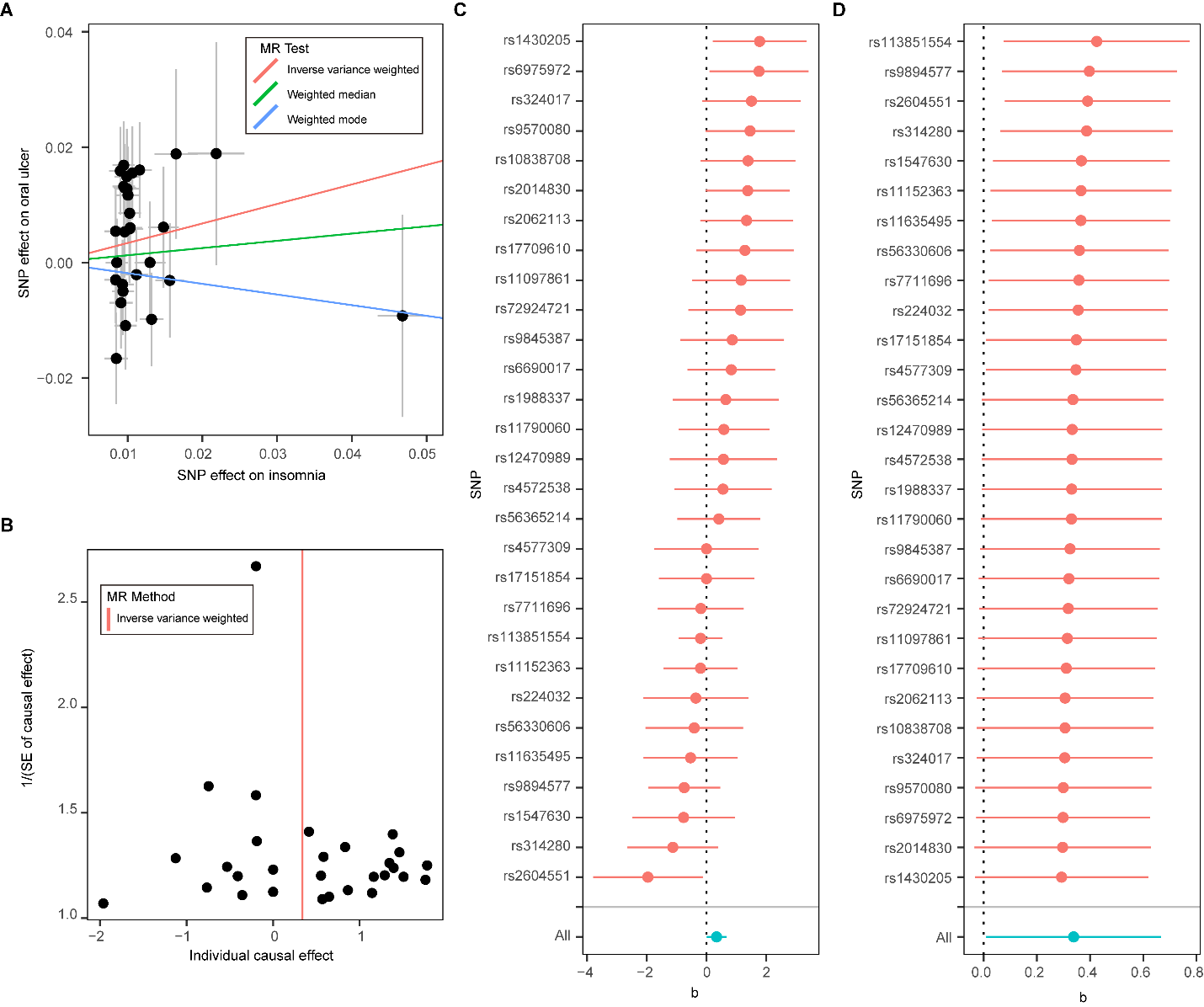
**Supplementary Figure 4. MR analysis results for** **insomnia on risk of** **mouth ulcer.**

**(A)** Scatter plot of genetic associations with insomnia (horizontal lines) against genetic associations with mouth ulcer (vertical lines). Error bars for genetic associations are 95% confidence intervals. The slopes of each line in the scatter plot represent the causal association for each method.

**(B)** Funnel plot of single-SNP effect estimates and corresponding inverse standard errors.

**(C)** Forest plot of the association of individual SNPs with sleep duration and oral cavity cancer, together with pooled estimates.

**(D)** Forest plot of the results of the leave-one-out sensitivity analysis, where each SNP in the instrument was iteratively removed from the instrumental variables.


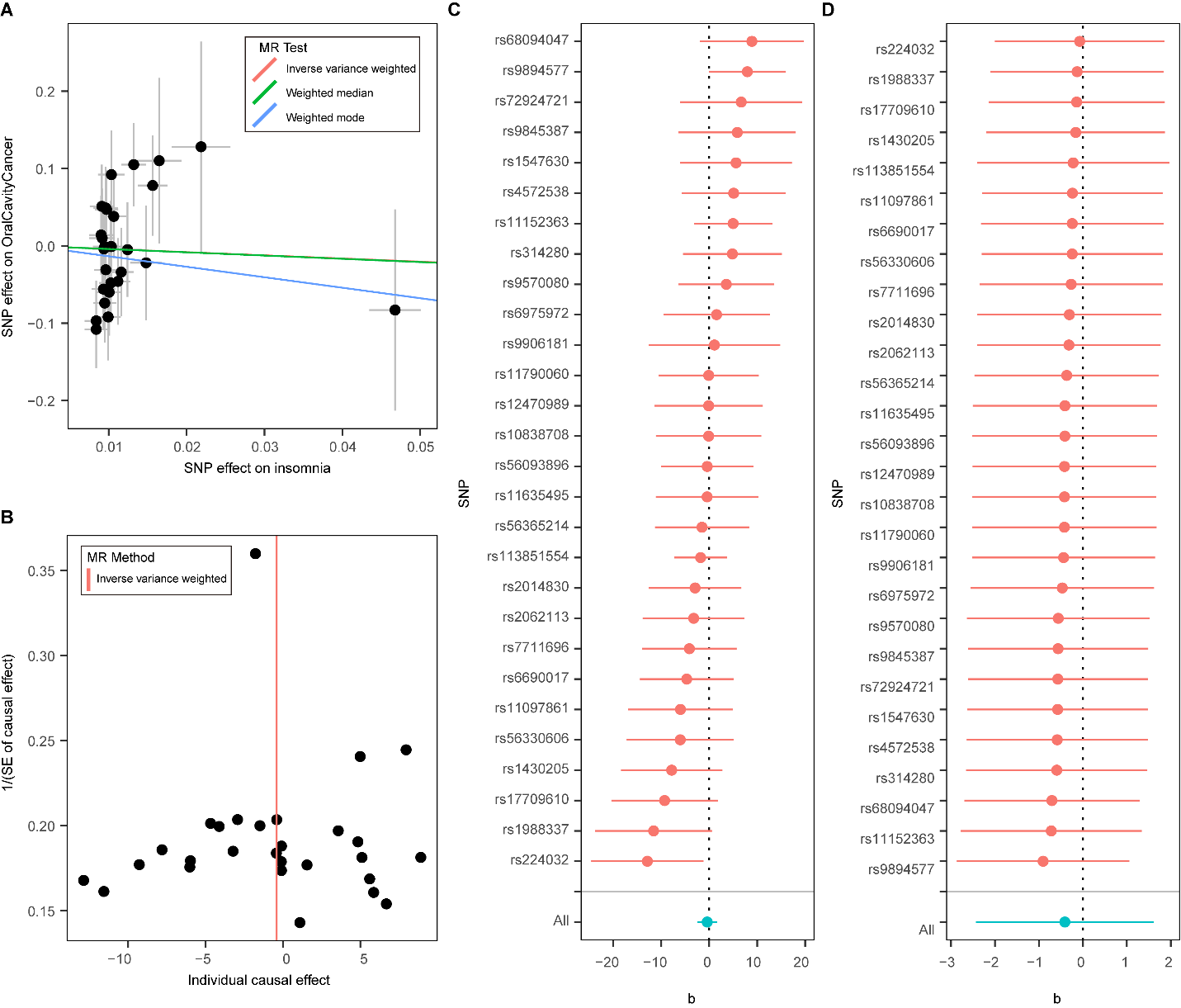
**Supplementary Figure 5. MR analysis results for insomnia on risk of** **oral cavity cancer.**

**(A)** Scatter plot of genetic associations with insomnia (horizontal lines) against genetic associations with oral cavity cancer (vertical lines). Error bars for genetic associations are 95% confidence intervals. The slopes of each line in the scatter plot represent the causal association for each method.

**(B)** Funnel plot of single-SNP effect estimates and corresponding inverse standard errors.

**(C)** Forest plot of the association of individual SNPs with sleep duration and oral cavity cancer, together with pooled estimates.

**(D)** Forest plot of the results of the leave-one-out sensitivity analysis, where each SNP in the instrument was iteratively removed from the instrumental variables.

**Supplementary Figure 6. MR analysis results for insomnia on risk of** **periodontal disease.**


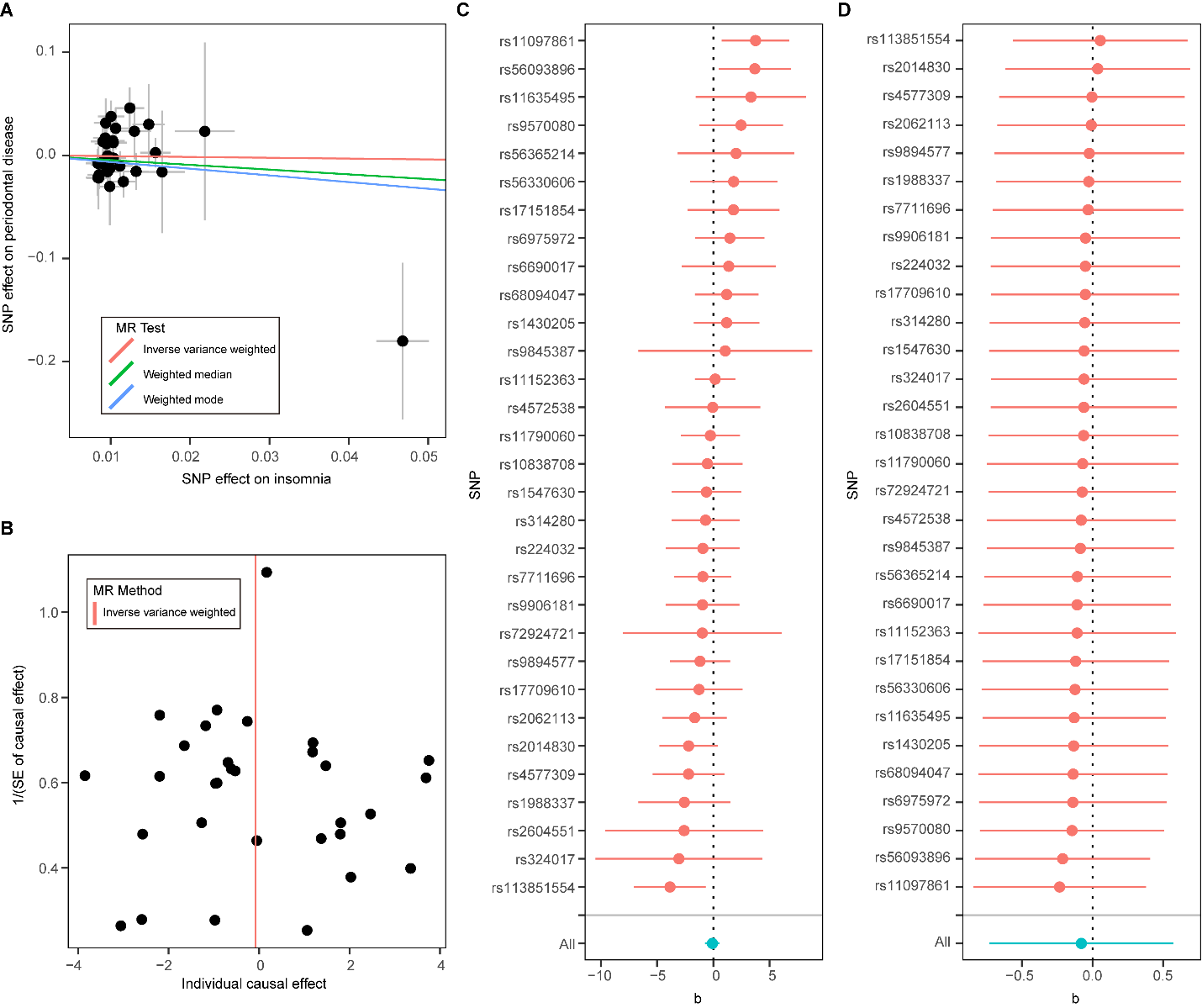


**(A)** Scatter plot of genetic associations with insomnia (horizontal lines) against genetic associations with periodontal disease (vertical lines). Error bars for genetic associations are 95% confidence intervals. The slopes of each line in the scatter plot represent the causal association for each method.

**(B)** Funnel plot of single-SNP effect estimates and corresponding inverse standard errors.

**(C)** Forest plot of the association of individual SNPs with sleep duration and oral cavity cancer, together with pooled estimates.

**(D)** Forest plot of the results of the leave-one-out sensitivity analysis, where each SNP in the instrument was iteratively removed from the instrumental variables.

**Supplementary Tables**

**Supplementary Table 1. Summary data from all GWAS used in current study.**

| Trait | Cases | Controls | Ethnic | Number of SNPs | PMID |
| --- | --- | --- | --- | --- | --- |
| Sleep duration | 460,099 | - | EUR | 9,851,867 | - |
| Insomnia | 462,341 | - | EUR | 9,851,867 | - |
| Mouth ulcer | 39,439 | 345,587 | EUR | 10,599,054 | 31427789 |
| Oral cavity cancer | 1,223 | 2,928 | EUR | 7,294,750 | 27749845 |
| Periodontal disease | 11,300 | 516,352 | EUR & EAS | 20,457,829 | 34594039 |

EUR, European; EAS, East Asian; SNP, single nucleotide polymorphism; GWAS, genome-wide association study; PMID, PubMed ID.

**Supplementary Table 2. Heterogeneity and horizontal pleiotropy analyses between insomnia, sleep duration and oral manifestations.**

| exposure trait | outcome trait | Heterogeneity | | | Horizontal pleiotropy | | | MR-PRESSO  P value |
| --- | --- | --- | --- | --- | --- | --- | --- | --- |
|  |  | IVW Q | IVW Q df | IVW P | Egger intercept | SE | P |  |
| Insomnia | Mouth ulcer | 40.32 | 28 | 0.06 | 5.68E-03 | 5.53E-03 | 0.31 | 0.07 |
|  | Oral cavity cancer | 30.78 | 27 | 0.28 | -3.87E-02 | 3.61E-02 | 0.29 | 0.28 |
|  | Periodontal disease | 36.48 | 30 | 0.19 | 1.12E-02 | 1.58E-02 | 0.48 | 0.20 |
| Sleep duration | Mouth ulcer | 60.29 | 50 | 0.15 | 3.89E-03 | 4.71E-03 | 0.41 | 0.16 |
|  | Oral cavity cancer | 56.07 | 45 | 0.12 | 7.19E-03 | 3.63E-02 | 0.84 | 0.11 |
|  | Periodontal disease | 63.52 | 56 | 0.23 | 2.15E-02 | 9.77E-03 | 0.03 | 0.22 |

IVW, Inverse variance weighted; Q, Cochran’s Q test estimate; df, Cochran’s Q test degrees of freedom; SE, standard error.

**Supplementary Table 3. Effect sizes can be detected with the power of 0.8 given the sample size, proportion of cases and variance explained by the instrumental variables.**

| exposure trait | outcome trait | sample size of outcome GWAS | proportion of cases in outcome GWAS | variance explained by instrumental variables | effect size |
| --- | --- | --- | --- | --- | --- |
| Insomnia | Mouth ulcer | 385,026 | 0.102 | 2.90E-03 | 0.25 |
|  | Oral cavity cancer | 4,151 | 0.295 | 3.61E-03 | 1.12 |
|  | Periodontal disease | 527,652 | 0.021 | 3.08E-03 | 0.40 |
| Sleep duration | Mouth ulcer | 385,026 | 0.102 | 4.82E-03 | 0.20 |
|  | Oral cavity cancer | 4,151 | 0.295 | 5.19E-03 | 0.99 |
|  | Periodontal disease | 527,652 | 0.021 | 6.42E-03 | 0.29 |
