# Supplementary material for: Identification of the causal relationship between sleep quality, insomnia, and oral ulcers
Source: BMC Oral Health. 2023 Oct 13;23:754. doi: 10.1186/s12903-023-03417-w (PMC10571295; doi:10.1186/s12903-023-03417-w)
Supplement: Supplementary file 2 — Supplementary Material 2 [file 12903_2023_3417_MOESM2_ESM.docx]

**STROBE-MR checklist of recommended items to address in reports of Mendelian randomization studies**^1^ ^2^

| **Item No.** | **Section** | **Checklist item** | **Page No.** | **Relevant text from manuscript** |
| --- | --- | --- | --- | --- |
| 1 | **TITLE and ABSTRACT** | Indicate Mendelian randomization (MR) as the study’s design in the title and/or the abstract if that is a main purpose of the study | 2 | We utilized genetic correlation and two-sample Mendelian randomization analyses based on summary statistics from genome-wide association studies |
|  | **INTRODUCTION** |  |  |  |
| 2 | **Background** | Explain the scientific background and rationale for the reported study. What is the exposure? Is a potential causal relationship between exposure and outcome plausible? Justify why MR is a helpful method to address the study question | 3-4 | The aim of the current study was to examine the causal relationship between sleep duration, insomnia, and the incidence of common oral diseases, specifically mouth ulcers, periodontal disease, and oral cavity cancer, using the two-sample MR method. The findings revealed a significant association between longer sleep duration and a lower risk of mouth ulcers, whereas a nominal association was identified between insomnia and increased risk of mouth ulcers. |
| 3 | **Objectives** | State specific objectives clearly, including pre-specified causal hypotheses (if any). State that MR is a method that, under specific assumptions, intends to estimate causal effects | 4 | Mendelian randomization (MR) is a useful causal inference method which could avoid common pitfalls in observational studies. The MR method uses genetic variation to infer causal association between modifiable exposures and health, developmental or social outcomes (13). By utilizing genetic variants as instrumental variables, the MR method provides a means to mitigate the impact of unobserved confounding factors on the relationship between exposure and outcome. |
|  | **METHODS** |  |  |  |
| 4 | **Study design and data sources** | Present key elements of the study design early in the article. Consider including a table listing sources of data for all phases of the study. For each data source contributing to the analysis, describe the following: | 4-5 | The GWAS summary datasets used in this study were listed in Supplementary Table 1. |
|  | a) | Setting: Describe the study design and the underlying population, if possible. Describe the setting, locations, and relevant dates, including periods of recruitment, exposure, follow-up, and data collection, when available. | 4 | A detailed description of the study design, including quality control procedures and statistical analyses, is available at http://www.nealelab.is/uk-biobank/. |
|  | b) | Participants: Give the eligibility criteria, and the sources and methods of selection of participants. Report the sample size, and whether any power or sample size calculations were carried out prior to the main analysis | 4 | The GWAS summary datasets used in this study were listed in Supplementary Table 1. Summary statistics of sleep duration were from a genome-wide association study (GWAS) based on the UK Biobank data (N=460,099). Sleep duration was measured by “About how many hours sleep do you get in every 24 hours” in the UK Biobank (data field: 1160). Summary statistics of insomnia were from GWAS based on the UK Biobank data (N=462,341). Insomnia was measured by “Do you have trouble falling asleep at night or do you wake up in the middle of the night?” in the UK Biobank (data field: 1200).  We analyzed three oral diseases as outcomes, including mouth ulcer (N=385,026) (14), periodontal disease (N=527,652) (15), and oral cavity cancer (N=4,151) (16), based on available summary statistics from previous GWAS. |
|  | c) | Describe measurement, quality control and selection of genetic variants | 5 | . Single nucleotide polymorphisms (SNP) with genome-wide significance threshold (P<5E-08) and in low linkage-disequilibrium with other SNPs (r2 < 0.001) within a clumping distance of 10,000 kb in each trait as exposure were selected as instrumental variables. Effects are harmonized to ensure that the effect estimates in both the exposure and outcome GWAS correspond to the same allele for each SNP. |
|  | d) | For each exposure, outcome, and other relevant variables, describe methods of assessment and diagnostic criteria for diseases | NA |  |
|  | e) | Provide details of ethics committee approval and participant informed consent, if relevant | 11 | Our study performed a secondary analysis of publicly available data, which was obtained with informed consent and ethical approvals from original GWAS studies. Therefore, no additional ethics approval or consent was necessary for our MR study. |
| 5 | **Assumptions** | Explicitly state the three core IV assumptions for the main analysis (relevance, independence and exclusion restriction) as well assumptions for any additional or sensitivity analysis | 6 | Three essential assumptions are prerequisites for conducting MR: (A) the relevance assumption, the instrumental variables are associated with the exposure; (B) the independence assumption, the instrumental variables are independent of any confounding factors of the exposure-outcome association; and (C) the exclusion-restriction assumption, the instrumental variables are conditionally independent of the outcome given the exposure and the confounding factors. |
| 6 | **Statistical methods: main analysis** | Describe statistical methods and statistics used | 6 | To evaluate the causative effect of sleep duration and insomnia on the risk of oral diseases, we performed a two-sample MR analysis using the random effects inverse variance weighted method. |
|  | a) | Describe how quantitative variables were handled in the analyses (i.e., scale, units, model) | NA |  |
|  | b) | Describe how genetic variants were handled in the analyses and, if applicable, how their weights were selected | 5 | Single nucleotide polymorphisms (SNP) with genome-wide significance threshold (P<5E-08) and in low linkage-disequilibrium with other SNPs (r2 < 0.001) within a clumping distance of 10,000 kb in each trait as exposure were selected as instrumental variables. Effects are harmonized to ensure that the effect estimates in both the exposure and outcome GWAS correspond to the same allele for each SNP. |
|  | c) | Describe the MR estimator (e.g. two-stage least squares, Wald ratio) and related statistics. Detail the included covariates and, in case of two-sample MR, whether the same covariate set was used for adjustment in the two samples | 6 | To evaluate the causative effect of sleep duration and insomnia on the risk of oral diseases, we performed a two-sample MR analysis using the random effects inverse variance weighted method.  We further verified the significant results using weighted median and weighted mode methods. |
|  | d) | Explain how missing data were addressed | NA |  |
|  | e) | If applicable, indicate how multiple testing was addressed | 6 | Bonferroni-corrected thresholds (0.05/6=8.33E-03) were adopted to account for multiple testing. |
| 7 | **Assessment of assumptions** | Describe any methods or prior knowledge used to assess the assumptions or justify their validity |  |  |
| 8 | **Sensitivity analyses and additional analyses** | Describe any sensitivity analyses or additional analyses performed (e.g. comparison of effect estimates from different approaches, independent replication, bias analytic techniques, validation of instruments, simulations) | 6 | To assess whether the MR assumptions were violated in the analysis, we conducted a number of sensitivity analyses. We calculated the F-statistic of each SNP, which could reflect the exact strength of the effect of SNPs on the exposure traits. SNPs with F statistic below 10 were considered as weak instruments and were thus removed. Multiple SNPs selected as instrumental variables are inevitably subjected to the pleiotropy issue. We utilized MR-PRESSO to test for horizontal pleiotropic outliers, and removed the outliers to reduce the effect of horizontal pleiotropy. Cochran’s Q statistic, which is derived from the IVW estimate, should follow a χ2 distribution with degrees of freedom equal to the number of SNPs minus 1. We performed Cochran’s Q test to check heterogeneity in the MR estimates. We further applied MR-Egger regression, a weighted linear regression of the SNP-outcome effects on the SNP-exposure effects allowing for the intercept to be estimated. The intercept provides a measure of average pleiotropic bias. The statistical power was calculated at http://cnsgenomics.com/shiny/mRnd/. |
| 9 | **Software and pre-registration** |  |  |  |
|  | a) | Name statistical software and package(s), including version and settings used | 6 | The R package TwoSampleMR 0.5.6 was used for the statistical analyses. |
|  | b) | State whether the study protocol and details were pre-registered (as well as when and where) | NA |  |
|  | **RESULTS** |  |  |  |
| 10 | **Descriptive data** |  |  |  |
|  | a) | Report the numbers of individuals at each stage of included studies and reasons for exclusion. Consider use of a flow diagram | NA |  |
|  | b) | Report summary statistics for phenotypic exposure(s), outcome(s), and other relevant variables (e.g. means, SDs, proportions) | 7 | Results showed that one standard deviation increase in genetically determined sleep duration was associated with a reduced risk of mouth ulcers (OR:0.67, 95% CI:0.54-0.83, P=2.84E-04) (Figure 1). Such association was further verified by the weighted median (OR: 0.61, 95% CI: 0.44-0.83, P =1.60E-03) and weighted mode (OR: 0.53, 95% CI: 0.30-0.95, P=0.037) methods. |
|  | c) | If the data sources include meta-analyses of previous studies, provide the assessments of heterogeneity across these studies | NA |  |
|  | d) | For two-sample MR:  i.  Provide justification of the similarity of the genetic variant-exposure associations between the exposure and outcome samples  ii.  Provide information on the number of individuals who overlap between the exposure and outcome studies | NA |  |
| 11 | **Main results** |  |  |  |
|  | a) | Report the associations between genetic variant and exposure, and between genetic variant and outcome, preferably on an interpretable scale | NA |  |
|  | b) | Report MR estimates of the relationship between exposure and outcome, and the measures of uncertainty from the MR analysis, on an interpretable scale, such as odds ratio or relative risk per SD difference | 7 | Results showed that one standard deviation increase in genetically determined sleep duration was associated with a reduced risk of mouth ulcers (OR:0.67, 95% CI:0.54-0.83, P=2.84E-04) (Figure 1). Such association was further verified by the weighted median (OR: 0.61, 95% CI: 0.44-0.83, P =1.60E-03) and weighted mode (OR: 0.53, 95% CI: 0.30-0.95, P=0.037) methods. |
|  | c) | If relevant, consider translating estimates of relative risk into absolute risk for a meaningful time period | NA |  |
|  | d) | Consider plots to visualize results (e.g. forest plot, scatterplot of associations between genetic variants and outcome versus between genetic variants and exposure) | 7 | Figure 1, Figure 2 and supplementary figures. |
| 12 | **Assessment of assumptions** |  |  |  |
|  | a) | Report the assessment of the validity of the assumptions | 7 | The funnel plot showed a visually apparent symmetry, which excluded the possible influence of directional pleiotropy on our estimates (Figure 2). |
|  | b) | Report any additional statistics (e.g., assessments of heterogeneity across genetic variants, such as *I^2^*, Q statistic or E-value) | 7 | We first estimated the genetic correlation between sleep duration, insomnia, and oral diseases. We detected a significant and negative genetic correlation between sleep duration and mouth ulcers (genetic correlation: -0.09, SE=0.03, P=0.007). Meanwhile, a significant and positive genetic correlation was identified between insomnia and mouth ulcers (genetic correlation: 0.18, SE=0.04, P=2.51E-06) (Supplementary Figure 1). |
| 13 | **Sensitivity analyses and additional analyses** |  |  |  |
|  | a) | Report any sensitivity analyses to assess the robustness of the main results to violations of the assumptions | 7 | Furthermore, we performed a number of sensitivity analyses to validate the causal association between sleep duration and the risk of oral diseases. No heterogeneity of effects between the instrumental variables was detected by the Cochran’s Q test (Supplementary Table 2). The F statistics of all the instrument variables were above 10 (ranging from 29 to 224), suggesting the selected instrumental variables were strong enough. The MR-Egger regression analysis gave no significant evidence of horizontal pleiotropy, as the intercept was not significantly deviated from zero (Supplementary Table 2). Meanwhile, no potential instrumental outlier was detected by the MR-PRESSO analysis (Supplementary Table 2). |
|  | b) | Report results from other sensitivity analyses or additional analyses | 7 | Same as above. |
|  | c) | Report any assessment of direction of causal relationship (e.g., bidirectional MR) | NA |  |
|  | d) | When relevant, report and compare with estimates from non-MR analyses | 7 | Same as above. |
|  | e) | Consider additional plots to visualize results (e.g., leave-one-out analyses) | 7 | Same as above. |
|  | **DISCUSSION** |  |  |  |
| 14 | **Key results** | Summarize key results with reference to study objectives | 8 | Our results showed that higher sleep duration was significantly associated with a reduced risk of mouth ulcers, whereas insomnia was nominally associated with an increased risk of mouth ulcers. |
| 15 | **Limitations** | Discuss limitations of the study, taking into account the validity of the IV assumptions, other sources of potential bias, and imprecision. Discuss both direction and magnitude of any potential bias and any efforts to address them | 10 | Firstly, despite our selection of genetic variants from large-scale studies, we cannot completely rule out the possibility of weak instrument bias, as is common in all MR analyses. Second, the results were obtained from Caucasian individuals and might not be generalized to other ethnic populations. Further replication based on cohorts of different ancestry was still warranted. Third, even though two-sample MR can be performed when the exposure of interest and the outcome are not simultaneously measured within one dataset, full datasets such as large-scale patient cohorts such as discovery and external validation sets are needed for a comprehensive understanding of causality, considering potential confounding factors. Fourth, the insomnia diagnosis and sleep time were collected based on the individuals’ subjective measurement, which might not be accurate and thus influence the results. |
| 16 | **Interpretation** |  |  |  |
|  | a) | Meaning: Give a cautious overall interpretation of results in the context of their limitations and in comparison with other studies | 8-10 | All relevant discussion. |
|  | b) | Mechanism: Discuss underlying biological mechanisms that could drive a potential causal relationship between the investigated exposure and the outcome, and whether the gene-environment equivalence assumption is reasonable. Use causal language carefully, clarifying that IV estimates may provide causal effects only under certain assumptions | 9 | One possible reason is that sleep duration could affect mouth ulcers by modulating immunologic response and inflammatory mediators (25). It was suggested that sleep deprivation increased serum TNF-α, IL-1β, IL-6, IL-8, and MCP-1 levels (10), while excessive production of TNF-α, IL-1β, and IL-6 were associated with an increased risk of recurrent aphthous stomatitis (26). Additionally, sleep deprivation induces oxidative stress in the body, which plays an important role in the oral mucosal disease pathogenesis (27). Therefore, inflammation and oxidative stress might be involved in the pathogenesis of how short sleep duration increased the risk of mouth ulcers. |
|  | c) | Clinical relevance: Discuss whether the results have clinical or public policy relevance, and to what extent they inform effect sizes of possible interventions | 10 | These findings have implications for informing therapeutic interventions and drug development in future clinical trials. |
| 17 | **Generalizability** | Discuss the generalizability of the study results (a) to other populations, (b) across other exposure periods/timings, and (c) across other levels of exposure | 10 | Second, the results were obtained from Caucasian individuals and might not be generalized to other ethnic populations. Further replication based on cohorts of different ancestry was still warranted. |
|  | **OTHER INFORMATION** |  |  |  |
| 18 | **Funding** | Describe sources of funding and the role of funders in the present study and, if applicable, sources of funding for the databases and original study or studies on which the present study is based | 11 | This work was supported by the National Natural Science Foundation of China (Grant No. 82002877, U19A2005, 82270986). |
| 19 | **Data and data sharing** | Provide the data used to perform all analyses or report where and how the data can be accessed, and reference these sources in the article. Provide the statistical code needed to reproduce the results in the article, or report whether the code is publicly accessible and if so, where | 11 | Summary statistics of sleep duration and insomnia could be downloaded from ieu open gwas project (https://gwas.mrcieu.ac.uk/, ID: ukb-b-4424 and ukb-b-3957). Summary statistics of periodontal disease and oral cavity cancer could be found in GWAS Catalog (ID: GCST90018897 and GCST012237). Summary statistics of mouth ulcer could be found in GWAS Atlas (https://atlas.ctglab.nl/traitDB/3544, atlas ID: 3544). |
| 20 | **Conflicts of Interest** | All authors should declare all potential conflicts of interest | 11 | The authors declare that they have no competing interests. |

This checklist is copyrighted by the Equator Network under the Creative Commons Attribution 3.0 Unported (CC BY 3.0) license.

1. Skrivankova VW, Richmond RC, Woolf BAR, Yarmolinsky J, Davies NM, Swanson SA, et al. Strengthening the Reporting of Observational Studies in Epidemiology using Mendelian Randomization (STROBE-MR) Statement. JAMA. 2021;under review.

2. Skrivankova VW, Richmond RC, Woolf BAR, Davies NM, Swanson SA, VanderWeele TJ, et al. Strengthening the Reporting of Observational Studies in Epidemiology using Mendelian Randomisation (STROBE-MR): Explanation and Elaboration. BMJ. 2021;375:n2233.
